# Development and Validation of an Instrument to Measure Career Decision-Making Challenges of International Medical Students in China

**DOI:** 10.5334/pme.1384

**Published:** 2024-11-22

**Authors:** Wen Li, Asaduzzaman Khan, Robyn Gillies, Hong Sun

**Affiliations:** 1 School of Education, Faculty of Humanities, Arts and Social Sciences, The University of Queensland, Brisbane, QLD 4072, Australia; 2 School of Health and Rehabilitation Sciences, Faculty of Health and Behavioural Sciences, The University of Queensland, Brisbane, QLD 4072, Australia; 3 School of Basic Medicine, Xuzhou Medical University, Xuzhou 221004, China

## Abstract

**Introduction:**

International medical students (IMSs) experience various problems preventing them from making career decisions. Assessing the difficulties involved in the career decision-making process is instrumental for identifying the sources of their career indecision, which may assist them in making more informed career decisions. This study aims to develop and validate an instrument to measure career decision-making challenges of IMSs in China, who are mainly from low- and middle-income countries.

**Methods:**

A new scale, INternational meDical studEnt Carrer decISION-making Scale (INDECISION Scale) was developed utilising data from IMSs in China. Initial item generation stemmed from a literature review and qualitative interviews (n = 20), with items adapted or formulated referencing phrasing used in prior instruments. Subsequent expert validation and cognitive interviews (n = 6) informed adjustments, followed by a pilot study (n = 52) and focus group discussions (n = 6). Exploratory factor analysis (EFA) was performed on data from four Chinese universities (n = 334), followed by confirmatory factor analysis (CFA) on data from eight other Chinese universities (n = 514). Convergent validity (n = 102) and test-retest reliability (n = 86) were evaluated using subsets of respondents.

**Results:**

The EFA retained 21 items, identifying six factors: unreadiness; lack of self-knowledge; lack of options knowledge; external complexity; lack of decision-making competence; and negative mentality. The CFA confirmed the six-factor model, demonstrating satisfactory model fit indices. Convergent validity and test-retest reliability were supported.

**Conclusions:**

The INDECISION Scale exhibits adequate psychometric properties, helping IMSs systematically navigate their decision-making process, allowing for individual challenges to be effectively identified for discussion in counselling. This study serves as a starting point for further research on career indecision and career guidance for IMSs.

## Introduction

Medical students have been reported to encounter diverse challenges in navigating the process of making career decisions [1]. These challenges serve as sources of career indecision, delaying firm decisions or leading to suboptimal choices [2]. With uncertain career intentions, many medical students may change their career plans or specialties during medical school [3,4], hindering goal setting and career preparation [5]. Some graduates may later regret their initial choices, altering their specialty during residency or even in practice [1,4,6], with negative effects on career development. Assessing these challenges is essential for identifying the causes of indecision and offering tailored guidance to support career planning [7], potentially reducing future disappointments and improving public health [2,8].

Career indecision is one of the core research issues in vocational psychology, with considerable effort dedicated to developing and validating tools to assess difficulties that impede the career decision-making process [9]. A systematic review identified 27 instruments measuring challenges in career decision-making, highlighting key sources of career indecision such as lack of readiness, orientation, or information, and misuse of information [2]. Despite this, this issue has received limited attention in medical career research [10]. Only a handful of studies have used these tools with medical students: An and Lee [5] analysed relationships between career indecision, social support, career exploration behaviour and career decision-making self-efficacy in Korea using the Korean Career Barrier Inventory; Chen et al. [8] applied the Career Factors Inventory to study career indecision and associated factors in Malaysia; Zhu et al. [10] employed the Career Decision-Making Difficulties Questionnaire (CDDQ) in China, exploring sources of career indecision and its links to coping strategies and well-being; and Alibhai et al. [11] used the CDDQ to evaluate a career intervention in Canada. However, these instruments, developed for general populations, may be less relevant for medical students.

One instrument specifically designed to measure medical students’ specialty indecision is the Specialty Indecision Scale (SIS) [1]. The SIS was conceptualised and designed based on the US medical training system, where medical education is at the graduate level and medical students are regarded as upper-level professional students who need to select a specialty from their already chosen career [4]. An updated version of the SIS which integrated other previous theoretical frameworks was published in the Manual for the Specialty Indecision Scale and is accessible on the Association of American Medical Colleges’ Careers in Medicine website [4]. Several studies have employed either the original or updated version of the SIS to assess medical specialty indecision, with the main purposes to evaluate the effectiveness of a career course intervention [12], to analyse the relationship between career maturity and specialty indecision [7], or to explore the factors associated with subspecialty indecision during residency training [6].

The global shortage of doctors is a widespread concern, particularly pressing in low- and middle-income countries (LMICs) [13]. With the internationalisation of medical education, a rising number of students opt to study medicine abroad, including those from LMICs [14]. Currently, about 68,000 international medical students (IMSs), primarily from LMICs in Africa and Asia, are studying in Chinese universities [14]. Given their substantial numbers and potential contributions to healthcare in both their home countries and internationally [15], understanding their career decision-making is crucial. IMSs also reportedly encounter indecision in career decision making [13], and cross-border transitions add further challenges [15]. Therefore, it is pivotal to provide IMSs with a suitable tool to gauge the sources of their indecision [16], allowing for effective identification and addressing of these challenges through appropriate career support. However, our literature review found no studies using such tools to investigate IMSs’ career decision-making challenges.

For medical students, including IMSs, choosing a specialty and practice location are key aspects of career decision-making [17]. For IMSs, migration after graduation is equally, if not more, important [13]. In some countries, medical students also face the decision of whether to stay in the medical profession [18]. Given IMSs’ diverse backgrounds and the complexity of their career choices, it is reasonable to use an instrument that broadly addresses indecision across the overall career decision-making process, rather than focusing on just one aspect.

In light of the relevance of comprehending the sources of career indecision among IMSs and the absence of a suitable measuring instrument, we endeavoured to design and validate a new tool by extending and adapting prior work with new items specific to the IMSs’ context. This tool can help education institutions assess the challenges IMSs face and improve career services. It also allows IMSs to better understand their own difficulties in making career decisions, enabling them to seek personalised support from faculty and career professionals for more informed choices.

## Methods

### Study context and design

The extensive application and relevance of the SIS (2nd edition) among medical school population has been vital to shaping and creating the current instrument [4]. However, given that over 15 years have passed since its publication and IMSs may encounter specific issues related to their context of studying in a different country, the INternational meDical studEnt Carrer decISION-making Scale (INDECISION Scale) was designed and validated following the best practice guidelines for scale development and validation [19,20]. This process enabled us to verify the existing challenges in prior measurements as well as identify new issues among the population of IMSs in current time. The scale development and scale validation were delineated across two phases, detailed in Appendix 1.

In particular, we intended to examine the convergent validity between this new instrument and the well-established SIS [4]. If desirable correlation between the two measures and dimensions could be estimated, convincing evidence for validity of the new instrument could be better supported [19]. Additionally, IMSs’ SIS ratings were compared to those of native medical students from previous studies, aiding in the identification of areas reflecting the specific needs of IMSs.

This study was conducted among IMSs in China for three main reasons. First, the large and diverse IMS population in China provided a robust participant pool. Second, a significant proportion of IMSs in China hail from LMICs, aligning with our target demographic and offering insights to address the critical shortage of medical professionals in these regions. Third, two authors (WL and HS) have over ten years of experience with international medical programmes in China, leveraging their familiarity with the context and networks to secure consent from gatekeepers in the respective universities. This strategic selection ensured optimal conditions for the study [21].

### Ethical considerations

Ethics approval was obtained from The University of Queensland, Australia (2022/HE001071). Approvals to conduct the research at the participating universities were obtained through the gatekeepers in written or oral form adhering to the pertinent rules and regulations of each respective institution. Written consents were obtained from the participants involved in interviews or focus group discussions. Implied consents were obtained from the survey participants.

### Phase 1: Procedure of scale development

#### Literature review

A literature review was conducted to elucidate the dimensions of career indecision and identify existing measurement tools for medical students [20]. We conducted a systematic search in PubMed, Scopus, CINAHL, and ERIC, using keywords related to medical students and career indecision, covering literature from 1980 to 2022. The search strategy for one database (PubMed) is presented in Appendix 2. The execution of our screening procedures referred to the PRISMA guidelines [22]. Studies focusing on career indecision among medical students were included, while those limited to career choices or career preferences were excluded. Eleven articles were retained [3,4,5,6,7,8,10,11,12,23,24]. Content analysis by the first author (WL) [25] identified five major dimensions and 11 sub-dimensions aligned with the categorisation from Kulcsár et al. [2] (Appendix 3). Classic career theories were reviewed for item development guidance [26,27].

#### Qualitative study

Semi-structured interviews with IMSs were guided by the Cognitive Information Processing (CIP) theory [28] to explore career decision-making challenges. Six themes emerged (Appendix 3). Many challenges aligned with prior literature [14], reflecting common issues among medical students across cultures. Meanwhile, unique issues specific for IMSs were also identified, emphasising the need for more tailored items in the instrument to capture their particular concerns. Full study details were published elsewhere [14].

#### Analysis and synthesis

Insights from interviews and the literature were compared to reconcile the constructs, laying the groundwork for questionnaire item generation. Key components of the CIP theory, including the Pyramid of Information Processing Model and Decision-Making Readiness Model, were integrated with the literature review dimensions and qualitative themes (Appendix 3). Affective elements such as anxious feelings and psychological distress were embedded into the scale [29,30]. By incorporating a sound theoretical framework in the item development, the instrument’s design was enhanced and potential shortcomings were minimised [31]. This process led to the development of seven domains.

#### Item generation

One researcher (WL) drafted items based on the integrated summarisation from the previous step, referencing phrasing from prior instruments [1,4,32]. Following the SIS (2nd edition) [4], as well as the SIS (1st edition) [1] and the CDDQ [32], on which the SIS (2nd edition) was based, items were written or adapted to better fit the context of IMSs, with full considerations of its broad scope, wide applicability and parsimonious wording (Appendix 1). The team reviewed and revised the draft, resulting in 34 items across seven domains (Appendix 4).

#### Expert validation

Three experts with rich experience in international medical programmes independently reviewed the 34 items using a content validity form template suggested by Allen et al. [33]. Based on their feedback, 20 items remained unchanged, two were reworded, and two were removed. Additionally, all three experts advised shortening the scale to reduce cognitive burden [19,34]. This led to a more concise item set, improving factor analysis sample size and data generalisability [35]. Items with similar meanings were combined to be more generic [33]. This process yielded a refined set of 27 items (Appendix 4).

#### Cognitive interview

We conducted cognitive interviews with six IMSs with diverse demographics [19], using think-aloud and verbal probing techniques [20]. Participants were requested to paraphrase the items, explain their responses, and offer feedback on the items, resulting in six reworded items after thematic analysis (Appendix 4).

#### Pilot study

We conducted a pilot study with 60 IMSs to assess the distribution of participants’ responses on the items [20], gathering 52 valid responses. Using an electronic questionnaire, we included a free-text section for feedback on the items and additional career decision-making challenges. Freshmen and sophomores noted that “I’m overwhelmed with clinical/internship duties to start career decision making” was irrelevant to those without clinical exposure. Based on this feedback, we combined this item with a similar item, resulting in the updated statement: “I’m overwhelmed with the study burden or internship duties to consider career decision making”.

#### Focus group discussion

We conducted two focus group discussions with six IMSs from the pilot study to review the results, focusing on items with a standard deviation lower than 1.0 [36] or a skewed response distribution [37]. Students raised concerns about the item “I face extra uncertainties and problems created by the COVID-19 pandemic”, suggesting its removal due to the diminishing impact of the pandemic at the time of this discussion. This was confirmed by the second group, leading to its deletion. No further changes were made, and the formal survey was finalised (Appendix 5).

#### Time 1 survey measurement

Given that our scale was newly developed, we opted for exploratory factor analysis (EFA) in the initial phase to uncover its underlying factor structure [35]. The INDECISION Scale was administered online at four universities across different regions of China to collect evidence for psychometric item and structure analysis [36]. The survey link was shared with all available classes of IMSs across all study years through social network class groups by the administrators in international medical programmes at these universities. Responses were analysed using IBM SPSS Statistics (Version 29). The EFA was performed by using the principal components extraction method with Varimax rotation.

### Phase 2: Procedure of scale validation

#### Time 2 survey measurement

To validate the scale, we evaluated the EFA-informed factor structure and tested the fit validity via the confirmatory factor analysis (CFA) [35], using a different sample of IMSs. The survey was administered at eight universities located in seven administrative provincial regions in China. The survey link was sent to all available classes of IMSs across all study years via social network class groups by administrators or teaching staff engaged in international medical programmes at the respective universities. Participants were asked about their willingness to join a follow-up survey, with email addresses collected confidentially for this purpose.

We used AMOS software for CFA with the maximum likelihood estimation method to evaluate the model fit. The path coefficients between an item and its EFA-informed dimension needed to be statistically significant (P < 0.05). Modification indices guided the identification of additional theoretically meaningful paths for improved model fit [38]. The model fit was assessed by using various absolute, incremental, and predictive fit indices with reference to the desired cutoff values for the respective solutions [38,39,40]. The desirable cutoff values are presented in Table 3.

#### Time 3 survey measurement

We collected evidence of construct validity of the scale via convergent validity and estimated the reliability of the scale via test-retest reliability [41], by using data from the Time 3 survey measurement, which was administered within a subset of the sample one week after the Time 2 survey administration. This follow-up survey included the INDECISION Scale for the test-retest reliability analysis and the SIS [4] for convergent validity analysis. A short interval was chosen to minimise the effect of ongoing academic and practical experiences on retest results [41].

Participants for the Time 3 survey were selected based on their availability and willingness, providing email addresses for the follow-up. The follow-up survey link was emailed individually seven (7) days after their Time 2 survey completion, with a requested three-day response window, ensuring a retest interval of 7–10 days. We confirmed with the institutions that no career-related activities were held for IMSs during this time. Test-retest reliability was calculated using Pearson correlation of the scores on the instrument at two occasions, and adequate reliability was demonstrated by a correlation coefficient greater than 0.70 [42].

The SIS [4] was used as a comparator instrument to test the convergent validity, as it was designed to measure indecision for specialty decision making of medical students and thus also provided information for challenges during the career decision making process [1]. Despite the INDECISION Scale assessing overall career indecision and the SIS focusing on specialty indecision, we hypothesised that these two measures would be positively related due to their perceived connection. Additionally, as all the items on the two scales were associated with difficulties during career decision-making, we hypothesised any dimension on the INDECISION Scale would be positively related to any dimension on the SIS [38]. Convergent validity was estimated by the Pearson correlation using SPSS, with coefficients categorised as very strong (0.90–1.00), strong (0.70–0.89), moderate (0.40–0.69), or weak (0.10–0.39) correlations [43].

## Results

### Participant characteristics

In the Time 1 survey, out of 408 individuals who clicked on the survey link, 334 were included after excluding those lacking informed consent or being incomplete, resulting in an inclusion rate of 81.9%. This sample was deemed sufficient for the recommended sample size to perform EFA [44]. In the Time 2 survey, out of 585 clicks, 514 complete responses with informed consent were included, yielding an inclusion rate of 87.9%. Table 1 shows participant characteristics, and the sample was representative of the IMS nationality composition in China [13], with no statistically significant demographic or career certainty differences between the EFA and CFA samples.

**Table 1:** Participants’ characteristics to INDECISION Scale by factor analysis type.


CHARACTERISTIC	EFA, N (% OF 334)	CFA, N (% OF 514)	P-VALUE^a^

**Gender**			

Male	179 (53.6%)	268 (52.1%)	.679

Female	155 (46.4%)	246 (47.9%)	

**Year of study** ^b^			

Pre-clinical year	137 (41.0%)	208 (40.5%)	.545

Clinical year	133 (39.8%)	192 (37.4%)	

Internship year	64 (19.2%)	114 (22.2%)	

**Country of origin**			

Asian LMIC^c^	287 (85.9%)	438 (85.2%)	.606^d^

African LMIC^c^	39 (11.7%)	64 (12.5%)	

Oceanian LMIC^c^	0 (0%)	4 (0.8%)	

South American LMIC^c^	1 (0.3%)	1 (0.2%)	

HIC^c^	7 (2.1%)	7 (1.4%)	

**Career certainty**			

Certain	304 (91.0%)	455 (88.5%)	.246^e^

Uncertain	30 (9.0%)	59 (11.5%)	


Note: ^a^ Chi-square test was employed. ^b^ As IMSs in China started to have clinical courses from their fourth year of study, the responses from first, second and third years of study were grouped into preclinical year, the responses from fourth and fifth years of study were grouped into clinical year, and responses in internship year were from the IMSs who had completed five years of courses and were doing/prepared to do the internship. The number of participants from first year to internship year: EFA, 45, 40, 52, 54, 79, 64; CFA, 49, 77, 82, 69, 123, 114. ^c^ LMIC is abbreviation for low- and middle-income country; HIC is abbreviation for high-income country. ^d^ Chi-square test was based on three groups. Group 1: Asian LMIC; Group 2: African, Oceanian, and South American LMIC; Group 3: HIC. ^e^ Certain group included those who selected very certain and certain; Uncertain group included those who selected very uncertain and uncertain.

For the Time 3 follow-up, among the 285 respondents expressing willingness to be contacted, we received 102 responses via email. Their responses for both the INDECISION Scale and the SIS on this survey were complete, making it possible for us to include all the 102 responses for the convergent validity assessment. For the test-retest reliability analysis, we excluded responses that couldn’t be linked to previous surveys or showed major changes in career certainty [37]. Of the 102 responses, 88 were matched via email addresses, and after further screening for career certainty changes, 86 responses were included in the test-retest reliability analysis.

### EFA results

The Cronbach’s Alpha of 0.926 indicated a high level of internal consistency for our scale with this sample. The Kaiser–Meyer–Olkin (KMO) Measure of Sampling Adequacy was 0.910, showing that EFA was applicable. The Bartlett’s Test of Sphericity was significant (p < 0.001), suggesting patterned relationship between the items [44]. The factor structure was determined by using the Kaiser’s criterion (eigenvalues greater than 1.0), the scree plot, and the interpretability of the factor structure [45]. To select the most representative items, we applied the deletion criteria: (a) item with factor loadings below 0.4 [35] and (b) item with cross-loadings with a difference of less than 0.2 [46].

Six factors were extracted (Appendix 6). Based on the item deletion criteria, four items were dropped. One item (I have concerns about bias from potential employers) did not load above 0.4 on any factors. The intended dimension of negative affection was not identified as a distinctive factor in EFA. However, two of the items about negative affection loaded above 0.4 on the factors of unreadiness and negative thinking. Three items had cross-loadings with a difference of less than 0.2 (Appendix 6). After deleting the four items, we ran the EFA again to confirm the factor structure and to ensure all the retained items did not meet the deletion criteria [47]. This six-factor solution was interpretable and had theoretical senses [37]. Subject to this result, we altered the name of the negative thinking factor into negative mentality factor to enhance the accuracy of the factor name. We concluded that the 21 items were retained with the six-factor structure, accounting for 77.5% of the total variance. The factor loadings of the retained items and Cronbach’s Alpha coefficient of each dimension demonstrated good initial validity and reliability of the dimensions in this measure [35] (Table 2).

**Table 2:** EFA factor loadings and Cronbach’s alpha coefficient (after item deletion) (n = 334).


ITEMS	FACTOR LOADINGS AFTER VARIMAX ROTATION						CRONBACH’S ALPHA

	1	2	3	4	5	6	

UR1. I’m overwhelmed with the study burden or internship duties to consider career decision making	.849						.820

UR2. I feel unwilling to start the process of making career decisions	.793						

UR3. I don’t know where to begin, because there are too many options and factors to consider	.761						

LSK1. I need to know more about my goal		.875					.924

LSK2. I need to know more about my personality		.862					

LSK3. I need to know more about my capability		.850					

LSK4. I need to know more about my interests		.605					

LOK1. I lack information about where and from whom I can seek career guidance resources.			.847				.890

LOK2. I encounter challenges in obtaining information regarding the recognition of overseas medical degrees			.820				

LOK3. I need more clinical experience to gather information about career-related characteristics			.811				

LOK4. It’s hard for me to get adequate and reliable information about career options			.729				

EC1. I have financial concerns for the desired career				.863			.791

EC2. I face extra procedures or disadvantages related to overseas medical education				.781			

EC3. There is disagreement between me and someone important to me on my desired career				.742			

LDMC1. I’m hesitant among two or more career options					.846		.857

LDMC2. I’m of two minds towards the desired career					.819		

LDMC3. Making decisions is always hard for me					.707		

NM1. I think about obstacles a lot						.848	.875

NM2. I’m anxious about making a career choice						.813	

NM3. I doubt my competence in achieving the desired career goals						.783	

NM4. I question whether the choice made by myself is the right choice						.564	


Notes: ^a^ Factor loadings above 0.40 are reported. ^b^ Abbreviations: UR represents Unreadiness; LSK, Lack of self-knowledge; LOK, Lack of options knowledge; EC, External complexity; LDMC, Lack of decision-making competence; NM, Negative mentality.

### CFA results

**Figure 1:**
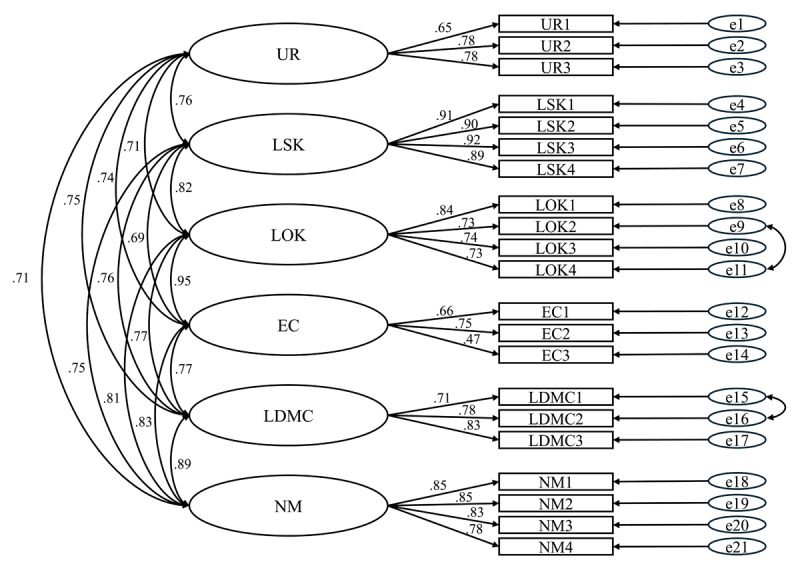
CFA model of INDECISION Scale. Legend: The values presented with the arrows represent correlation coefficients.

All items loaded significantly on the EFA-informed factors (P < 0.05), with the standardised regression coefficients ranging from 0.47 to 0.92 (Figure 1). According to the modification indices generated from the CFA, two minor additional paths that were theoretically meaningful and contributed to a better model fit were identified. These two additional paths were: 1) the correlation between error terms for two items in the lack of options knowledge factor (LOK2 and LOK4), and 2) the correlation between error terms for two items in the lack of decision-making competence factor (LDMC1 and LDMC2) (Figure 1). The value of Cronbach’s alpha was 0.955, indicating good internal consistency.

The CFA fit indices of the six-factor model of INDECISION Scale are shown in Table 3. The Normed χ^2^ statistic of 3.431 indicated an acceptable goodness of model fit [39,48]. All the incremental fit indices were above the target value 0.90, and the GFI (0.899) was very close to 0.90 [38]. The RMSEA and SRMR were both within acceptable levels (below 0.08) [40]. Overall, the model fit indices indicated a good fit model.

**Table 3:** CFA fit indices of the six-factor model of INDECISION Scale (n = 514).


FIT INDICES	FIT INDICES DEMONSTRATING GOODNESS-OF-FIT	MEASURED VALUES

**Absolute fit indices**		

Normed χ^2^ (χ^2^/*df*)	desired range of values: 2–5	3.431(χ^2^ goodness of fit: 590.121; *df*: 172)

Root mean square error of approximation (RMSEA)	<.08	.069

Standardised root mean square residual (SRMR)	<.08	.046

Goodness-of-fit index (GFI)	>.90	.899

**Incremental fit indices**		

Normed fit index (NFI)	>.90	.929

Comparative fit index (CFI)	>.90	.948

Tucker–Lewis index (TLI)	>.90	.937

**Predictive fit index**		

Expected cross-validation index (ECVI)	the default model value < the independence model value	Default: 1.380 Saturated: .901 Independence: 16.173

**Akaike information criterion (AIC)**	the hypothesized model value < the independence model value	Default: 708.121 Saturated: 462.000 Independence: 8296.851


Notes: ^a^ Factor loadings above 0.40 are reported. ^b^ Abbreviations: UR represents Unreadiness; LSK, Lack of self-knowledge; LOK, Lack of options knowledge; EC, External complexity; LDMC, Lack of decision-making competence; NM, Negative mentality.

### Convergent validity assessment

The correlation coefficient matrix of all dimensions for the INDECISION Scale and the SIS is presented in Appendix 7. Generally, the correlation coefficients indicated positive, moderate, and statistically significant correlations of the dimensions on the INDECISION Scale with those on the SIS. Additionally, a strong, positive correlation (r = 0.768, P < 0.001) between the two entire measures was observed. Our hypotheses regarding convergent validity were thus supported.

### Test-retest reliability assessment

The test-retest correlation coefficients for all dimensions on the INDECISION Scale at two occasions are presented in Appendix 8. The test-retest correlation coefficient of the entire measure (r = 0.831, p < 0.001) exhibited adequate test-retest reliability. The test-retest correlation coefficients for the six dimensions were all statistically significant (p < 0.001), ranging between 0.683 and 0.759, among which all but one (External complexity) were above 0.70.

### Comparison of scores among the SIS and INDECISION Scale

Appendix 9 shows IMSs’ ratings for each dimension on the SIS and the INDECISION Scale in the current study, alongside corresponding SIS ratings from non-IMSs in prior studies for comparison. On the SIS, IMSs ranked Information and Readiness higher than non-IMSs in both two previous studies. Additionally, when comparing IMSs’ ratings on the INDECISION Scale and the SIS, four out of six dimensions in the former ranked and scored higher than their corresponding dimensions in the latter (Information, Readiness, Self-Doubt, Barriers).

## Discussion

Our study addresses a notable gap in the existing research landscape by focusing on the career decision-making process of IMSs, an area largely overlooked. The absence of such a suitable instrument to assess their challenges underscores the necessity and urgency of our contribution. Following the SIS, we developed and validated the INDECISION Scale, based on CIP theory, literature reviews and qualitative interviews. Our findings demonstrate the scale’s reliability and validity in understanding sources of IMSs’ career indecision, using a robust sample from multiple institutions.

The INDECISION Scale was designed to guide IMSs in their career decision-making process by providing valuable insights through their ratings. The scale identifies dimensions that align with established constructs for career decision-making difficulties [2], while also including items specifically addressing the challenges unique to IMSs. According to Osipow and Winer [49], measuring career indecision serves as a pre-counselling experience that raises awareness and helps clarify indecision sources, facilitating the formal counselling process that follows. The new scale allows IMSs to systematically navigate their career decision-making, with ratings providing useful information that supports tailored guidance and a detailed exploration of individual challenges during counselling sessions. Career services for IMSs in China [14] and in other countries [15] are reportedly insufficient, and career counsellors tend to feel less competent in working with individuals who are culturally different [50]. The INDECISION Scale could serve as a foundation for improving these services, providing a framework for counsellors to guide discussions with IMSs [49]. Additionally, the scale can help institutions gather IMS-specific needs to design more targeted career guidance programmes and better prepare counsellors.

The strong overall correlation and moderate dimension correlation between the INDECISION Scale and SIS indicate a close link between IMSs’ indecision in specialty choice and their indecision in overall career choice. However, it is important to note that other aspects, such as practice location, practice type, and academic responsibilities, can also add challenges [13,17]. Given their visa limitations, IMSs may face additional stress regarding migration decisions, making guidance on these aspects crucial [14]. Therefore, identifying the indecision sources and clarifying which aspects this indecision is towards are essential for more effective interpretation and use of the measurement in counselling.

Comparing SIS ratings between IMSs and non-IMSs from US [4] and Korean [7] studies showed IMSs placed greater emphasis on challenges in Information and Readiness dimensions. This heightened demand for career information and resources further reveals the underdevelopment of career guidance for IMSs, while their escalated difficulties in finding motivation to begin the process may be partially attributed to the added burden of managing dual curricula due to studying abroad, as noted in qualitative interviews [14]. On the other side, discrepancies in IMSs’ rankings and scores between the INDECISION Scale and SIS suggest the potential of the items on the new scale to capture issues relevant to IMSs. On the new scale, items explicitly referencing overseas medical degrees/education (LOK2, EC2) likely contribute to higher ratings in those dimensions, while some other items incorporating key points from IMSs’ qualitative interviews may enhance the relevance and elevate corresponding scores and rankings. Items containing internship duties (UR1) and clinical experience (LOK3) convey IMSs’ concerns, particularly clinical practice in context of the COVID-19 pandemic and accommodation issues for internships outside China [14]. IMSs’ higher rating on Negative Mentality dimension may be linked to the newly added items on negative thinking (NM1) and career choice doubt (NM4), possibly reflecting a generally more pessimistic outlook among IMSs or repercussion from the pandemic, such as prolonged online learning and restrictive policies towards online medical students [14]. These findings illustrate IMSs’ unique needs, including balancing academic and career planning, securing clinical opportunities, managing mental stress, and accessing improved career resources and counselling services.

Test-retest reliability showed adequate correlation, though slightly lower than expected, particularly for the External Complexity dimension (below 0.70). Mean scores dropped across all dimensions in the retest, suggesting reduced career decision-making challenges despite no career-related activities at school. This may reflect natural learning or increased awareness from the initial survey. These findings indicate the importance of identifying career indecision sources for IMSs and support the potential for a self-help mode in career services, aligning with the CIP-based differentiated service delivery model proposed by Osborn et al [51].

Since career indecision is an under-researched topic in medical education, it is hoped that this new instrument can serve as a starting point for further research. Future studies could assess its applicability across different cultures and medical student groups. Developed within the context of China, the scale could be tested and validated internationally, with necessary adjustments made to reflect the specific characteristics of medical education systems, medical practices, and cultural nuances of different countries. Moreover, studies can be conducted to compare the ratings between IMSs and native medical students, through interpreting the results of which, the accuracy and specificity of the items can be enhanced, consequently benefiting career guidance with more tailored insights. Given evidence that career stress and career uncertainty may vary with year of study and are possibly related to the timing of internships [8,52,53], further grade-stratified comparative analyses are recommended to explore the influence of study year on career indecision, aiding in instrument validation or refinement.

## Limitations

Our study has limitations. First, the possible influence of year of study on career indecision is not focused on in the current study, which should be considered when interpreting the findings. Second, due to limited literature on measurement of career indecision or career decision-making difficulties among medical students, some early references cited in our study may not fully capture the current landscape, which further indicates the research gap and highlights the need for more research on this topic. Third, the instrument is based on the context of IMSs in China, so further investigations in diverse international contexts with varied IMS compositions would enhance the instrument’s applicability and provide broader perspectives. As such, more items may be added to each dimension and retested, to further verify and improve the reliability and validity of the instrument across different cultural contexts.

## Conclusions

In conclusion, we have designed and validated an assessment tool to measure the career decision-making challenges among IMSs, addressing a gap in instruments for understanding career indecision among this student population. This study focuses on measuring IMSs’ overall career indecision from a broad perspective. The developed items may serve as a practical approach to help IMSs systematically navigate their decision-making, enabling effective identification of individual challenges for discussion in counselling. We hope this study contributes to facilitating the development of career guidance for IMSs and provides useful information for counselling practice and research.

## Data Accessibility Statement

Data supporting this study cannot be made available due to ethical restrictions.

## Additional File

The additional file for this article can be found as follows:

10.5334/pme.1384.s1 Supplementary Files. Appendixes 1 to 9.
