# Supplementary material for: Development and Validation of an Instrument to Measure Career Decision-Making Challenges of International Medical Students in China
Source: Perspect Med Educ. 2024 Nov 22;13(1):572–84. doi: 10.5334/pme.1384 (PMC11583610; doi:10.5334/pme.1384)
Supplement: Supplementary Files. — Appendixes 1 to 9. [file pme-13-1-1384-s1.zip › pme-1384_li-s1/Appendix 1.docx]

**Appendix 1** Scale development and validation procedure of the INDECISION Scale

| **Phase** | **Step** | **Participant** | **Data collection** | **Action** | **Strategy/Criteria** | **Results** |
| --- | --- | --- | --- | --- | --- | --- |
| **Scale development** | Literature review | 4 researchers |  | Scoping search and systematic search | Databases: PubMed, Scopus, CINAHL, and ERIC  Keywords: medical student, career indecision, career uncertain*, career barrier, career undecided*, career indecisiv*, career difficult*, career concern  Search strategy: Appendix 2.  Inclusion criteria: (1) empirical studies pertinent to difficulties and challenges faced by medical students in any aspect in career decision-making; (2) medical students at both undergraduate and postgraduate levels; (3) published in English language; (4) published between1980 and 2022  Exclusion criteria: (1) studies mainly addressing career choices or career preferences; (2) not published in English language; (3) not published between1980 and 2022 | 11 articles selected  5 major dimensions and 11 sub-dimensions summarised (Appendix 3) |
|  | Qualitative study | 20 IMSs | Purposeful sampling from one Chinese medical university | Conducting semi-structured interviews | Guiding framework: the Cognitive Information Processing (CIP) theory  Participants: 20 IMSs from 10 different countries studying in China  Sampling strategy: purposeful sampling based on gender, study year, and home country to ensure sample diversity  Initial coding strategy: the three fundamental concepts of knowledge, decision-making skills, and metacognition in the CIP pyramid structure  Data analysis: directed qualitative content analysis | 6 themes yielded (Appendix 3) |
|  | Analysis and synthesis of the findings | 4 researchers |  | Merging the findings from literature review and qualitative study into a comprehensive summarisation | Comparison of interview and literature insights, with integration of the key CIP theory components | 7 domains designed (Appendix 3) |
|  | Item generation | 4 researchers |  | Reviewing and discussing within the entire research team to reach consensus | Item were drafted based three considerations:  (1) Broad scope: The items were adapted to not focus on specialty indecision only but rather on the broad career perspective, based on IMSs’ simultaneous considerations of multiple career aspects, and their particular concerns on migration choice.  (2) Wide applicability: The items were formulated with full considerations of applicability among IMSs with diverse backgrounds.  (3) Parsimonious wording: As there would be an instruction for the IMSs that they were the targeted population for this scale and they would be directed to rate the items based on their own context, terms like “international medical student” or “overseas medical education” might not appear on each item. | 34 items formulated (Appendix 4) |
|  | Expert validation | 3 experts | Inviting experts with extensive experience in international medical programme in China | Providing feedback on items on content validity form | Expert panel: three experts with over ten years of experience of administration, teaching, and/or guidance in international medical programme in China were invited to independently review our initial items  Content validity form template: including domain’s definition, item list, domain assignments and a free-text comment section  Experts’ suggestions: (1) 20 items were agreed to remain unchanged, while 14 items were recommended for rewording, combination or removal by at least one expert. (2) All three experts advised shortening the scale to reduce participants’ cognitive burden. In line with expert feedback and Allen et al.’s criteria, items with similar or overlapping meanings were combined to be more generic | 20 items retained without change  2 items removed  2 items reworded  10 items combined into 5 generic ones  Totalling to 27 items  (Appendix 4) |
|  | Cognitive interview | 6 IMSs | Purposeful sampling from one Chinese medical university | Paraphrasing items, explaining responses, offering feedback on items | Sampling strategy: purposeful sampling  Techniques: Using think-aloud and verbal probing techniques recommended by Artino et al. [20], participants were prompted to articulate their thinking processes while answering each item. The interviewer interjected with specific questions at opportune moments to gain insights, trying utmost to not cause much disruption [20]. | 6 items reworded  (Appendix 4) |
|  | Pilot study | 52 IMSs | Purposeful sampling from one Chinese medical university | Doing online survey and providing additional challenges or feedback on the current items | Enrolment strategy: We invited 60 IMSs employing purposeful strategy for this pilot study in terms of gender, nationality, and year of study, by utilising the student list with the permission from the participants’ university.  Survey administration: online survey | 2 items combined into 1 item  Totalling to 26 items  (Appendix 4) |
|  | Focus group discussion | 6 IMSs | Selected from respondents in the pilot study | Providing in-depth opinions on the pilot study results | Sampling strategy: We conducted two focus group discussions with three IMSs in each group, who had participated in the pilot study  Focus of the items: items with a standard deviation lower than 1.0 and those showing apparently unbalanced response distribution (e.g. below 20% agreement or disagreement). This facilitated decisions on potential item adjustments to enhance response variability | 1 item removed  Totalling to 25 items  (Appendix 5) |
|  | Time 1 survey measurement | 334 IMSs (out of 408 clicks) | All available classes of IMSs at 4 Chinese universities, inclusion rate of 81.9% | Doing the INDECISION Scale for EFA analysis | Survey administration: online survey  Location of the participating universities: These four universities were situated in four administrative provincial regions dispersed in different geographical locations across China, with one in east, one in north, one in southwest and one in northwest. | 6 factors extracted  4 items removed  Totalling to 21 items  (Appendix 6)  (Table 2) |
| **Scale validation** | Time 2 survey measurement | 514 IMSs (out of 585 clicks) | All available classes of IMSs at another 8 Chinese universities, inclusion rate of 87.9% | Doing the INDECISION Scale for CFA analysis | Survey administration: online survey  Location of the participating universities: The eight universities were located in seven administrative provincial regions in China, with two regions in east, two in southwest, one in southeast, one in south and one in north | 6-factor solution confirmed  A good fit model  (Table 3) |
|  | Time 3 survey measurement | 102 IMSs | A subset of the respondents from time 2 survey measurement, who volunteered to participate | Doing the SIS for convergent validity assessment | Enrolment strategy: The subset was selected based on the respondents’ indication of availability in participation and willingness of providing their email addresses.  Management: The email address and the survey completion time of the participants who agreed to the follow-up survey were recorded into a spreadsheet.  Convergent validity was estimated by the Pearson correlation using SPSS, with coefficients categorised as very strong (0.90-1.00), strong (0.70-0.89), moderate (0.40-0.69), or weak (0.10-0.39) correlations  Hypotheses: (1) We hypothesised that the INDECISION Scale and the SIS would be positively related due to their perceived connection. (2) We hypothesised that any dimension on the INDECISION Scale would be positively related to any dimension on the SIS | Acceptable convergent validity  (Appendix 7) |
|  |  | 86 IMSs | A subset of the respondents from time 2 survey measurement, who volunteered to participate as well as provided identical email addresses in both Time 2 and Time 3 survey measurements | Doing the INDECISION Scale again for test-retest reliability assessment | Enrolment: The subset was selected based on the respondents’ indication of availability in participation and willingness of providing their email addresses.  Management: The email address and the survey completion time of the participants who agreed to the follow-up survey were recorded into a spreadsheet.  Test-retest reliability: correlation coefficient greater than 0.70  Exclusion criteria: (1) responses which were not able to be linked with those in the previous survey, and (2) responses whose career certainty changed from being certain to being uncertain or vice versa | Acceptable test-retest reliability  (Appendix 8) |
