# Supplementary material for: Development and Validation of an Instrument to Measure Career Decision-Making Challenges of International Medical Students in China
Source: Perspect Med Educ. 2024 Nov 22;13(1):572–84. doi: 10.5334/pme.1384 (PMC11583610; doi:10.5334/pme.1384)
Supplement: Supplementary Files. — Appendixes 1 to 9. [file pme-13-1-1384-s1.zip › pme-1384_li-s1/Appendix 1.pdf]

Appendix 1 Scale development and validation procedure of the INDECISION Scale

Phase	Step	Participant	Data collection	Action	Strategy/Criteria	Results
**Scale development**	Literature review	4 researchers		Scoping search and systematic search	Databases: PubMed, Scopus, CINAHL, and ERIC<br>Keywords: medical student, career indecision, career uncertain*, career barrier, career undecided*, career indecisiv*, career difficult*, career concern<br>Search strategy: Appendix 2.<br>Inclusion criteria: (1) empirical studies pertinent to difficulties and challenges faced by medical students in any aspect in career decision-making; (2) medical students at both undergraduate and postgraduate levels; (3) published in English language; (4) published between1980 and 2022<br>Exclusion criteria: (1) studies mainly addressing career choices or career preferences; (2) not published in English language; (3) not published between1980 and 2022	11 articles selected<br>5 major dimensions and 11 sub-dimensions summarised (Appendix 3)
	Qualitative study	20 IMSs	Purposeful sampling from one Chinese medical university	Conducting semi-structured interviews	Guiding framework: the Cognitive Information Processing (CIP) theory<br>Participants: 20 IMSs from 10 different countries studying in China<br>Sampling strategy: purposeful sampling based on gender, study year, and home country to ensure sample diversity	6 themes yielded (Appendix 3)

					Initial coding strategy: the three fundamental concepts of knowledge, decision-making skills, and metacognition in the CIP pyramid structure<br>Data analysis: directed qualitative content analysis	
	Analysis and synthesis of the findings	4 researchers		Merging the findings from literature review and qualitative study into a comprehensive summarisation	Comparison of interview and literature insights, with integration of the key CIP theory components	7 domains designed (Appendix 3)
	Item generation	4 researchers		Reviewing and discussing within the entire research team to reach consensus	Item were drafted based three considerations:<br>(1) Broad scope: The items were adapted to not focus on specialty indecision only but rather on the broad career perspective, based on IMSs' simultaneous considerations of multiple career aspects, and their particular concerns on migration choice.<br>(2) Wide applicability: The items were formulated with full considerations of applicability among IMSs with diverse backgrounds.<br>(3) Parsimonious wording: As there would be an instruction for the IMSs that they were the targeted population for this scale and they would be directed to rate the items based on their own context, terms like "international medical student"	34 items formulated (Appendix 4)

					or “overseas medical education” might not appear on each item.	
	Expert validation	3 experts	Inviting experts with extensive experience in international medical programme in China	Providing feedback on items on content validity form	Expert panel: three experts with over ten years of experience of administration, teaching, and/or guidance in international medical programme in China were invited to independently review our initial items<br>Content validity form template: including domain’s definition, item list, domain assignments and a free-text comment section<br>Experts’ suggestions: (1) 20 items were agreed to remain unchanged, while 14 items were recommended for rewording, combination or removal by at least one expert. (2) All three experts advised shortening the scale to reduce participants’ cognitive burden. In line with expert feedback and Allen et al.’s criteria, items with similar or overlapping meanings were combined to be more generic	20 items retained without change<br>2 items removed<br>2 items reworded<br>10 items combined into 5 generic ones<br>Totalling to 27 items<br>(Appendix 4)
	Cognitive interview	6 IMSs	Purposeful sampling from one Chinese medical university	Paraphrasing items, explaining responses, offering feedback on items	Sampling strategy: purposeful sampling<br>Techniques: Using think-aloud and verbal probing techniques recommended by Artino et al. [20], participants were prompted to articulate their thinking processes while answering each item. The interviewer interjected with specific questions at opportune moments to gain insights, trying utmost	6 items reworded<br>(Appendix 4)

					to not cause much disruption [20].	
	Pilot study	52 IMSs	Purposeful sampling from one Chinese medical university	Doing online survey and providing additional challenges or feedback on the current items	Enrolment strategy: We invited 60 IMSs employing purposeful strategy for this pilot study in terms of gender, nationality, and year of study, by utilising the student list with the permission from the participants' university.<br>Survey administration: online survey	2 items combined into 1 item<br>Totalling to 26 items<br>(Appendix 4)
	Focus group discussion	6 IMSs	Selected from respondents in the pilot study	Providing in-depth opinions on the pilot study results	Sampling strategy: We conducted two focus group discussions with three IMSs in each group, who had participated in the pilot study<br>Focus of the items: items with a standard deviation lower than 1.0 and those showing apparently unbalanced response distribution (e.g. below 20% agreement or disagreement). This facilitated decisions on potential item adjustments to enhance response variability	1 item removed<br>Totalling to 25 items<br>(Appendix 5)
	Time 1 survey measurement	334 IMSs (out of 408 clicks)	All available classes of IMSs at 4 Chinese universities, inclusion rate of 81.9%	Doing the INDECISION Scale for EFA analysis	Survey administration: online survey<br>Location of the participating universities: These four universities were situated in four administrative provincial regions dispersed in different geographical locations across China, with one in east, one in north, one in southwest and one in northwest.	6 factors extracted<br>4 items removed<br>Totalling to 21 items<br>(Appendix 6)<br>(Table 2)
**Scale validation**	Time 2 survey	514 IMSs (out of 585	All available classes of IMSs	Doing the INDECISION Scale	Survey administration: online survey<br>Location of the participating universities: The eight	6-factor solution confirmed

	measurement	clicks)	at another 8 Chinese universities, inclusion rate of 87.9%	for CFA analysis	universities were located in seven administrative provincial regions in China, with two regions in east, two in southwest, one in southeast, one in south and one in north	A good fit model (Table 3)
	Time 3 survey measurement	102 IMSs	A subset of the respondents from time 2 survey measurement, who volunteered to participate	Doing the SIS for convergent validity assessment	Enrolment strategy: The subset was selected based on the respondents' indication of availability in participation and willingness of providing their email addresses.<br>Management: The email address and the survey completion time of the participants who agreed to the follow-up survey were recorded into a spreadsheet.<br>Convergent validity was estimated by the Pearson correlation using SPSS, with coefficients categorised as very strong (0.90-1.00), strong (0.70-0.89), moderate (0.40-0.69), or weak (0.10-0.39) correlations<br>Hypotheses: (1) We hypothesised that the INDECISION Scale and the SIS would be positively related due to their perceived connection. (2) We hypothesised that any dimension on the INDECISION Scale would be positively related to any dimension on the SIS	Acceptable convergent validity (Appendix 7)
		86 IMSs	A subset of the respondents	Doing the INDECISION Scale	Enrolment: The subset was selected based on the respondents' indication of availability in	Acceptable test-retest

			from time 2 survey measurement, who volunteered to participate as well as provided identical email addresses in both Time 2 and Time 3 survey measurements	again for test-retest reliability assessment	participation and willingness of providing their email addresses.<br>Management: The email address and the survey completion time of the participants who agreed to the follow-up survey were recorded into a spreadsheet.<br>Test-retest reliability: correlation coefficient greater than 0.70<br>Exclusion criteria: (1) responses which were not able to be linked with those in the previous survey, and (2) responses whose career certainty changed from being certain to being uncertain or vice versa	reliability (Appendix 8)
