# Supplementary material for: Development and Validation of an Instrument to Measure Career Decision-Making Challenges of International Medical Students in China
Source: Perspect Med Educ. 2024 Nov 22;13(1):572–84. doi: 10.5334/pme.1384 (PMC11583610; doi:10.5334/pme.1384)
Supplement: Supplementary Files. — Appendixes 1 to 9. [file pme-13-1-1384-s1.zip › pme-1384_li-s1/Appendix 2.pdf]

Appendix 2 PubMed Search Strategy

Search: **medical student** Filters: **from 1980 - 2022**
("students, medical"[MeSH Terms] OR ("students"[All Fields] AND "medical"[All Fields]) OR "medical students"[All Fields] OR ("medical"[All Fields] AND "student"[All Fields]) OR "medical student"[All Fields]) AND (1980:2022[pdat])

165,285

AND

Search: **career indecision OR career uncertain* OR career barrier OR career undecided* OR career indecisiv* OR career difficult* OR career concern** Filters: **from 1980 - 2022**
((("career"[All Fields] OR "careers"[All Fields]) AND ("indecision"[All Fields] OR "indecisions"[All Fields] OR "indecisive"[All Fields] OR "indecisiveness"[All Fields])) OR (("career"[All Fields] OR "careers"[All Fields]) AND "uncertain*"[All Fields]) OR (("career"[All Fields] OR "careers"[All Fields]) AND ("barrier"[All Fields] OR "barrier s"[All Fields] OR "barriers"[All Fields])) OR (("career"[All Fields] OR "careers"[All Fields]) AND "undecided*"[All Fields]) OR (("career"[All Fields] OR "careers"[All Fields]) AND "indecisiv*"[All Fields]) OR (("career"[All Fields] OR "careers"[All Fields]) AND "difficult*"[All Fields]) OR (("career"[All Fields] OR "careers"[All Fields]) AND ("concern"[All Fields] OR "concerned"[All Fields] OR "concerning"[All Fields] OR "concerns"[All Fields]))) AND (1980:2022[pdat])

10,413

Total 1,761
