# Supplementary material for: Development and Validation of an Instrument to Measure Career Decision-Making Challenges of International Medical Students in China
Source: Perspect Med Educ. 2024 Nov 22;13(1):572–84. doi: 10.5334/pme.1384 (PMC11583610; doi:10.5334/pme.1384)
Supplement: Supplementary Files. — Appendixes 1 to 9. [file pme-13-1-1384-s1.zip › pme-1384_li-s1/Appendix 3.docx]

**Appendix 3** Scale domains combined with qualitative themes and literature review dimensions

| **Scale domain** | **CIP theory component** | **Qualitative theme** | **Literature review dimension** |
| --- | --- | --- | --- |
| External complexity | Readiness Model-complexity concerns | Facing contextual complexities that limit the career decision-making process | External conflicts |
| Unreadiness | Readiness Model-capability concerns | Feeling unwilling to begin the process of career decision making | Dysfunctional career beliefs |
| Negative affection |  |  | Lack of willingness |
|  | Pyramid Model-thinking about one’s own decision making | Being negative about career decision making | Anxiety |
| Negative thinking |  |  | Lack of confidence |
| Lack of decision-making competence | Pyramid Model-knowing how to make decisions | Lacking career decision-making skills | Lack of information about how to make career decisions |
|  |  |  | Indecisiveness |
|  |  |  | Internal conflicts |
| Lack of self-knowledge | Pyramid Model-knowing about oneself | Lacking knowledge about oneself | Lack of self information |
| Lack of options knowledge | Pyramid Model-knowing about one’s options | Lacking knowledge about career options | Lack of career information |
|  |  |  | Unreliable information |
