# Supplementary material for: Development and Validation of an Instrument to Measure Career Decision-Making Challenges of International Medical Students in China
Source: Perspect Med Educ. 2024 Nov 22;13(1):572–84. doi: 10.5334/pme.1384 (PMC11583610; doi:10.5334/pme.1384)
Supplement: Supplementary Files. — Appendixes 1 to 9. [file pme-13-1-1384-s1.zip › pme-1384_li-s1/Appendix 3.pdf]

Appendix 3 Scale domains combined with qualitative themes and literature review dimensions

Scale domain	CIP theory component	Qualitative theme	Literature review dimension
External complexity	Readiness Model-complexity concerns	Facing contextual complexities that limit the career decision-making process	External conflicts
Unreadiness	Readiness Model-capability concerns	Feeling unwilling to begin the process of career decision making	Dysfunctional career beliefs
Negative affection			Lack of willingness
	Pyramid Model-thinking about one's own decision making	Being negative about career decision making	Anxiety
Negative thinking			Lack of confidence
Lack of decision-making competence	Pyramid Model-knowing how to make decisions	Lacking career decision-making skills	Lack of information about how to make career decisions
			Indecisiveness
			Internal conflicts
Lack of self-knowledge	Pyramid Model-knowing about oneself	Lacking knowledge about oneself	Lack of self information
Lack of options knowledge	Pyramid Model-knowing about one's options	Lacking knowledge about career options	Lack of career information
			Unreliable information
