# Supplementary material for: Development and Validation of an Instrument to Measure Career Decision-Making Challenges of International Medical Students in China
Source: Perspect Med Educ. 2024 Nov 22;13(1):572–84. doi: 10.5334/pme.1384 (PMC11583610; doi:10.5334/pme.1384)
Supplement: Supplementary Files. — Appendixes 1 to 9. [file pme-13-1-1384-s1.zip › pme-1384_li-s1/Appendix 4.docx]

**Appendix 4** Item pool and actions taking place

| Domain | Initial items | Revised items | Action(s) taking place |
| --- | --- | --- | --- |
| Lack of self-knowledge | 1. I need to know more about my interests |  | Retained |
|  | 1. I need to know more about my capability |  | Retained |
|  | 1. I need to know more about my goal |  | Retained |
|  | 1. I need to know more about my personality |  | Retained |
|  | 1. I don’t know whether my personal characteristics suit for my desired career | I need to know more about my suitability for my desired career | Reworded (expert validation)  Removed (EFA) |
| Lack of options knowledge | 1. It’s hard to get adequate information about career | It’s hard for me to get adequate and reliable information about career options | Combined (expert validation) |
|  | 1. I can’t find reliable information about the career options |  |  |
|  | 1. I encounter challenges in obtaining information regarding the recognition of overseas medical degrees |  | Retained |
|  | 1. I lack clinical experience | I need more clinical experience to gather information about career-related characteristics | Reworded (expert validation) |
|  | 1. I lack information about the people who I can seek for career guidance | I lack information about where and from whom I can seek career guidance resources | Combined (expert validation) |
|  | 1. I lack information about where I can get resources of career guidance |  |  |
|  | 1. I don’t know how to find career information |  | Removed (expert validation) |
| External complexity | 1. There are extra procedures or disadvantages related to overseas medical education | I face extra procedures or disadvantages related to overseas medical education | Reworded (cognitive interview) |
|  | 1. The pandemic causes extra uncertainties and problems | I face extra uncertainties and problems created by the COVID-19 pandemic | Reworded (cognitive interview)  Removed (pilot study) |
|  | 1. I have financial concerns for the desired career |  | Retained |
|  | 1. There is disagreement between me and my family members on my desired career | There is disagreement between me and someone important to me on my desired career | Combined (expert validation) |
|  | 1. There is disagreement between me and my friends on my desired career |  |  |
|  | 1. I have concerns about bias from potential employers |  | Removed (EFA) |
| Lack of decision-making competence | 1. I am unsure about my desired career | I’m of two minds towards the desired career | Combined (expert validation)  Reworded (cognitive interview) |
|  | 1. I’m indecisive as I think my plan can change |  |  |
|  | 1. I can’t decide among some career options | I’m hesitant among two or more career options | Reworded (cognitive interview) |
|  | 1. I can’t decide as the two career options are equally attractive |  | Removed (expert validation) |
|  | 1. I’m an indecisive person in personality | Making decisions is always hard for me | Reworded (cognitive interview) |
| Unreadiness | 1. I’m unready because I’m too busy with my studies | I’m overwhelmed with clinical/internship duties to start career decision making (items 25 and 26 combined during expert validation)  I’m overwhelmed with the study burden or internship duties to consider career decision making (item 24 combined together during pilot study) | 25 & 26 combined (expert validation)  24 combined together (pilot study) |
|  | 1. I’m unready because I’m overwhelmed with practical classes |  |  |
|  | 1. I’m unready because I’m too busy with internship duties |  |  |
|  | 1. Complexities exist among my career choices so I don’t know where to start | I don’t know where to begin, because there are too many options and factors to consider | Reworded (cognitive interview) |
|  | 1. I’m unready to be honest in exploring myself |  | Removed (EFA) |
| Negative feeling | 1. I feel unwilling to start the process of making career decisions |  | Retained |
|  | 1. I feel stressful to accept the responsibility of the made choice |  | Removed (EFA) |
|  | 1. I’m anxious about making a career decision |  | Retained |
| Negative thinking | 1. I doubt my competence in achieving the desired career goals |  | Retained |
|  | 1. I think about obstacles a lot |  | Retained |
|  | 1. I question whether choice made by myself is the right choice |  | Retained |
