# Supplementary material for: Development and Validation of an Instrument to Measure Career Decision-Making Challenges of International Medical Students in China
Source: Perspect Med Educ. 2024 Nov 22;13(1):572–84. doi: 10.5334/pme.1384 (PMC11583610; doi:10.5334/pme.1384)
Supplement: Supplementary Files. — Appendixes 1 to 9. [file pme-13-1-1384-s1.zip › pme-1384_li-s1/Appendix 4.pdf]

Appendix 4 Item pool and actions taking place

Domain	Initial items	Revised items	Action(s) taking place
**Lack of self-knowledge**	1. I need to know more about my interests		Retained
	2. I need to know more about my capability		Retained
	3. I need to know more about my goal		Retained
	4. I need to know more about my personality		Retained
	5. I don't know whether my personal characteristics suit for my desired career	I need to know more about my suitability for my desired career	Reworded (expert validation)<br>Removed (EFA)
**Lack of options knowledge**	6. It's hard to get adequate information about career	It's hard for me to get adequate and reliable information about career options	Combined (expert validation)
	7. I can't find reliable information about the career options		
	8. I encounter challenges in obtaining information regarding the recognition of overseas medical degrees		Retained
	9. I lack clinical experience	I need more clinical experience to gather information about career-related characteristics	Reworded (expert validation)
	10. I lack information about the people who I can seek for career guidance	I lack information about where and from whom I can seek career guidance resources	Combined (expert validation)
	11. I lack information about where I can get resources of career guidance		
	12. I don't know how to find career information		Removed (expert validation)
**External complexity**	13. There are extra procedures or disadvantages related to overseas medical education	I face extra procedures or disadvantages related to overseas medical education	Reworded (cognitive interview)

	14. The pandemic causes extra uncertainties and problems	I face extra uncertainties and problems created by the COVID-19 pandemic	Reworded (cognitive interview)<br>Removed (pilot study)
	15. I have financial concerns for the desired career		Retained
	16. There is disagreement between me and my family members on my desired career	There is disagreement between me and someone important to me on my desired career	Combined (expert validation)
	17. There is disagreement between me and my friends on my desired career		
	18. I have concerns about bias from potential employers		Removed (EFA)
**Lack of decision-making competence**	19. I am unsure about my desired career	I'm of two minds towards the desired career	Combined (expert validation)<br>Reworded (cognitive interview)
	20. I'm indecisive as I think my plan can change		
	21. I can't decide among some career options	I'm hesitant among two or more career options	Reworded (cognitive interview)
	22. I can't decide as the two career options are equally attractive		Removed (expert validation)
	23. I'm an indecisive person in personality	Making decisions is always hard for me	Reworded (cognitive interview)
**Unreadiness**	24. I'm unready because I'm too busy with my studies	I'm overwhelmed with clinical/internship duties to start career decision making (items 25 and 26 combined during expert validation)<br>I'm overwhelmed with the study burden or internship duties to consider career decision making (item 24 combined together during pilot study)	25 & 26 combined (expert validation)<br>24 combined together (pilot study)
	25. I'm unready because I'm overwhelmed with practical classes		
	26. I'm unready because I'm too busy with internship duties		
	27. Complexities exist among my career choices so I don't know where to start	I don't know where to begin, because there are too many options and factors to consider	Reworded (cognitive interview)

	28. I'm unready to be honest in exploring myself		Removed (EFA)
**Negative feeling**	29. I feel unwilling to start the process of making career decisions		Retained
	30. I feel stressful to accept the responsibility of the made choice		Removed (EFA)
	31. I'm anxious about making a career decision		Retained
**Negative thinking**	32. I doubt my competence in achieving the desired career goals		Retained
	33. I think about obstacles a lot		Retained
	34. I question whether choice made by myself is the right choice		Retained
