# Supplementary material for: Development and Validation of an Instrument to Measure Career Decision-Making Challenges of International Medical Students in China
Source: Perspect Med Educ. 2024 Nov 22;13(1):572–84. doi: 10.5334/pme.1384 (PMC11583610; doi:10.5334/pme.1384)
Supplement: Supplementary Files. — Appendixes 1 to 9. [file pme-13-1-1384-s1.zip › pme-1384_li-s1/Appendix 5.docx]

**Appendix 5** INDECISION Scale for EFA

Q1. Please indicate your overall certainty towards your career intentions (including specialty, migration, location, practice type, and etc).

1. Very certain
2. Certain
3. Uncertain
4. Very uncertain

Q2. How much do you agree with the following statements describing your overall experience of career decision making (including specialty, migration, location, practice type, and etc)? Choose 1 if you strongly disagree and 5 if you strongly agree.

|  | Strongly disagree (1) | Disagree (2) | Neutral (3) | Agree (4) | Strongly agree (5) |
| --- | --- | --- | --- | --- | --- |
| 1. I’m overwhelmed with the study burden or internship duties to consider career decision making |  |  |  |  |  |
| 1. I don’t know where to begin, because there are too many options and factors to consider |  |  |  |  |  |
| 1. I’m unready to be honest in exploring myself |  |  |  |  |  |
| 1. I feel unwilling to start the process of making career decisions |  |  |  |  |  |
| 1. I feel stressful to accept the responsibility of the made choice |  |  |  |  |  |
| 1. I’m anxious about making a career decision |  |  |  |  |  |
| 1. I need to know more about my interests |  |  |  |  |  |
| 1. I need to know more about my capability |  |  |  |  |  |
| 1. I need to know more about my goal |  |  |  |  |  |
| 1. I need to know more about my personality |  |  |  |  |  |
| 1. I need to know more about my suitability for my desired career |  |  |  |  |  |
| 1. It’s hard for me to get adequate and reliable information about career options |  |  |  |  |  |
| 1. I encounter challenges in obtaining information regarding the recognition of overseas medical degrees |  |  |  |  |  |
| 1. I need more clinical experience to gather information about career-related characteristics |  |  |  |  |  |
| 1. I lack information about where and from whom I can seek career guidance resources |  |  |  |  |  |
| 1. I face extra procedures or disadvantages related to overseas medical education |  |  |  |  |  |
| 1. I have financial concerns for the desired career |  |  |  |  |  |
| 1. There is disagreement between me and someone important to me on my desired career |  |  |  |  |  |
| 1. I have concerns about bias from potential employers |  |  |  |  |  |
| 1. I’m of two minds towards the desired career |  |  |  |  |  |
| 1. I’m hesitant among two or more career options |  |  |  |  |  |
| 1. Making decisions is always hard for me |  |  |  |  |  |
| 1. I doubt my competence in achieving the desired career goals |  |  |  |  |  |
| 1. I think about obstacles a lot |  |  |  |  |  |
| 1. I question whether choice made by myself is the right choice |  |  |  |  |  |
