# Supplementary material for: Development and Validation of an Instrument to Measure Career Decision-Making Challenges of International Medical Students in China
Source: Perspect Med Educ. 2024 Nov 22;13(1):572–84. doi: 10.5334/pme.1384 (PMC11583610; doi:10.5334/pme.1384)
Supplement: Supplementary Files. — Appendixes 1 to 9. [file pme-13-1-1384-s1.zip › pme-1384_li-s1/Appendix 5.pdf]

Appendix 5 INDECISION Scale for EFA

Q1. Please indicate your overall certainty towards your career intentions (including specialty, migration, location, practice type, and etc).

A. Very certain
B. Certain
C. Uncertain
D. Very uncertain

Q2. How much do you agree with the following statements describing your overall experience of career decision making (including specialty, migration, location, practice type, and etc)? Choose 1 if you strongly disagree and 5 if you strongly agree.

	Strongly disagree (1)	Disagree (2)	Neutral (3)	Agree (4)	Strongly agree (5)
1. I’m overwhelmed with the study burden or internship duties to consider career decision making					
2. I don’t know where to begin, because there are too many options and factors to consider					
3. I’m unready to be honest in exploring myself					
4. I feel unwilling to start the process of making career decisions					
5. I feel stressful to accept the responsibility of the made choice					

6. I’m anxious about making a career decision					
7. I need to know more about my interests					
8. I need to know more about my capability					
9. I need to know more about my goal					
10. I need to know more about my personality					
11. I need to know more about my suitability for my desired career					
12. It’s hard for me to get adequate and reliable information about career options					
13. I encounter challenges in obtaining information regarding the recognition of overseas medical degrees					
14. I need more clinical experience to gather information about career-related characteristics					
15. I lack information about where and from whom I can seek career guidance resources					
16. I face extra procedures or disadvantages related to overseas medical education					
17. I have financial concerns for the desired career					

18. There is disagreement between me and someone important to me on my desired career					
19. I have concerns about bias from potential employers					
20. I’m of two minds towards the desired career					
21. I’m hesitant among two or more career options					
22. Making decisions is always hard for me					
23. I doubt my competence in achieving the desired career goals					
24. I think about obstacles a lot					
25. I question whether choice made by myself is the right choice					
