# Supplementary material for: Development and Validation of an Instrument to Measure Career Decision-Making Challenges of International Medical Students in China
Source: Perspect Med Educ. 2024 Nov 22;13(1):572–84. doi: 10.5334/pme.1384 (PMC11583610; doi:10.5334/pme.1384)
Supplement: Supplementary Files. — Appendixes 1 to 9. [file pme-13-1-1384-s1.zip › pme-1384_li-s1/Appendix 6.docx]

**Appendix 6** EFA factor loadings for six-factor solution of INDECISION Scale (n=334)

| **Items** | **Factor loadings after Varimax rotation** | | | | | |
| --- | --- | --- | --- | --- | --- | --- |
|  | **1** | **2** | **3** | **4** | **5** | **6** |
| I need to know more about my goal | .872 |  |  |  |  |  |
| I need to know more about my personality | .847 |  |  |  |  |  |
| I need to know more about my capability | .841 |  |  |  |  |  |
| I need to know more about my interests | .623 |  |  | .400 |  |  |
| I need to know more about my suitability for my desired career | .538 | .503 |  |  |  |  |
| I lack information about where and from whom I can seek career guidance resources. |  | .825 |  |  |  |  |
| I encounter challenges in obtaining information regarding the recognition of overseas medical degrees |  | .821 |  |  |  |  |
| I need more clinical experience to gather information about career-related characteristics |  | .782 |  |  |  |  |
| It’s hard for me to get adequate and reliable information about career options |  | .726 |  |  |  |  |
| I think about obstacles a lot |  |  | .839 |  |  |  |
| I’m anxious about making a career choice |  |  | .808 |  |  |  |
| I doubt my competence in achieving the desired career goals |  |  | .786 |  |  |  |
| I question whether choice made by myself is the right choice |  |  | .570 |  |  |  |
| I’m overwhelmed with the study burden or internship duties to consider career decision making |  |  |  | .804 |  |  |
| I don’t know where to begin, because there are too many options and factors to consider |  |  |  | .778 |  |  |
| I feel unwilling to start the process of making career decisions |  |  |  | .773 |  |  |
| I’m unready to be honest in exploring myself | .457 |  |  | .494 |  |  |
| I feel stressful to accept the responsibility of the made choice |  |  | .436 | .443 |  |  |
| I’m of two minds towards the desired career |  |  |  |  | .784 |  |
| I’m hesitant among two or more career options |  |  |  |  | .775 |  |
| Making decisions is always hard for me |  |  |  |  | .693 |  |
| I have financial concerns for the desired career |  |  |  |  |  | .834 |
| I face extra procedures or disadvantages related to overseas medical education |  |  |  |  |  | .779 |
| There is disagreement between me and someone important to me on my desired career |  |  |  |  |  | .739 |

Notes: ^a^ Factor loadings above 0.40 are reported.

^b^ Factor 1: Lack of self-knowledge; Factor 2: Lack of options knowledge; Factor 3: Negative mentality; Factor 4: Unreadiness; Factor 5: Lack of decision-making competence; Factor 6: External complexity
