# Supplementary material for: Development and Validation of an Instrument to Measure Career Decision-Making Challenges of International Medical Students in China
Source: Perspect Med Educ. 2024 Nov 22;13(1):572–84. doi: 10.5334/pme.1384 (PMC11583610; doi:10.5334/pme.1384)
Supplement: Supplementary Files. — Appendixes 1 to 9. [file pme-13-1-1384-s1.zip › pme-1384_li-s1/Appendix 6.pdf]

Appendix 6 EFA factor loadings for six-factor solution of INDECISION Scale (n=334)

Items	Factor loadings after Varimax rotation					
	1	2	3	4	5	6
I need to know more about my goal	.872					
I need to know more about my personality	.847					
I need to know more about my capability	.841					
I need to know more about my interests	.623			.400		
I need to know more about my suitability for my desired career	.538	.503				
I lack information about where and from whom I can seek career guidance resources.		.825				
I encounter challenges in obtaining information regarding the recognition of overseas medical degrees		.821				
I need more clinical experience to gather information about career-related characteristics		.782				
It’s hard for me to get adequate and reliable information about career options		.726				
I think about obstacles a lot			.839			
I’m anxious about making a career choice			.808			
I doubt my competence in achieving the desired career goals			.786			
I question whether choice made by myself is the right choice			.570			
I’m overwhelmed with the study burden or internship duties to consider career decision making				.804		
I don’t know where to begin, because there are too many options and factors to consider				.778		
I feel unwilling to start the process of making career decisions				.773		
I’m unready to be honest in exploring myself	.457			.494		
I feel stressful to accept the responsibility of the made choice			.436	.443		
I’m of two minds towards the desired career					.784	
I’m hesitant among two or more career options					.775	
Making decisions is always hard for me					.693	
I have financial concerns for the desired career						.834
I face extra procedures or disadvantages related to overseas medical education						.779
There is disagreement between me and someone important to me on my desired career						.739

Notes: [a] Factor loadings above 0.40 are reported.

[b] Factor 1: Lack of self-knowledge; Factor 2: Lack of options knowledge; Factor 3: Negative mentality; Factor 4: Unreadiness; Factor 5: Lack of decision-making competence; Factor 6: External complexity
