# Supplementary material for: Development and Validation of an Instrument to Measure Career Decision-Making Challenges of International Medical Students in China
Source: Perspect Med Educ. 2024 Nov 22;13(1):572–84. doi: 10.5334/pme.1384 (PMC11583610; doi:10.5334/pme.1384)
Supplement: Supplementary Files. — Appendixes 1 to 9. [file pme-13-1-1384-s1.zip › pme-1384_li-s1/Appendix 7.docx]

**Appendix 7** Correlation coefficient matrix for INDECISION Scale and SIS (n=102)

|  | I1 | I2 | I3 | I4 | I5 | I6 | IT | S1 | S2 | S3 | S4 | S5 | S6 | ST |
| --- | --- | --- | --- | --- | --- | --- | --- | --- | --- | --- | --- | --- | --- | --- |
| I1 | 1 |  |  |  |  |  |  |  |  |  |  |  |  |  |
| I2 | .749^**^ | 1 |  |  |  |  |  |  |  |  |  |  |  |  |
| I3 | .698^**^ | .773^**^ | 1 |  |  |  |  |  |  |  |  |  |  |  |
| I4 | .544^**^ | .692^**^ | .676^**^ | 1 |  |  |  |  |  |  |  |  |  |  |
| I5 | .602^**^ | .776^**^ | .603^**^ | .653^**^ | 1 |  |  |  |  |  |  |  |  |  |
| I6 | .591^**^ | .737^**^ | .727^**^ | .684^**^ | .728^**^ | 1 |  |  |  |  |  |  |  |  |
| IT | .807^**^ | .924^**^ | .882^**^ | .812^**^ | .838^**^ | .876^**^ | 1 |  |  |  |  |  |  |  |
| S1 | .657^**^ | .605^**^ | .520^**^ | .543^**^ | .637^**^ | .542^**^ | .671^**^ | 1 |  |  |  |  |  |  |
| S2 | .673^**^ | .653^**^ | .666^**^ | .547^**^ | .595^**^ | .663^**^ | .739^**^ | .743^**^ | 1 |  |  |  |  |  |
| S3 | .641^**^ | .650^**^ | .520^**^ | .522^**^ | .649^**^ | .590^**^ | .689^**^ | .823^**^ | .824^**^ | 1 |  |  |  |  |
| S4 | .488^**^ | .572^**^ | .441^**^ | .551^**^ | .597^**^ | .501^**^ | .604^**^ | .810^**^ | .756^**^ | .836^**^ | 1 |  |  |  |
| S5 | .600^**^ | .638^**^ | .509^**^ | .571^**^ | .699^**^ | .610^**^ | .697^**^ | .768^**^ | .731^**^ | .797^**^ | .739^**^ | 1 |  |  |
| S6 | .700^**^ | .723^**^ | .586^**^ | .546^**^ | .664^**^ | .672^**^ | .755^**^ | .750^**^ | .835^**^ | .812^**^ | .707^**^ | .789^**^ | 1 |  |
| ST | .696^**^ | .709^**^ | .601^**^ | .604^**^ | .708^**^ | .662^**^ | .768^**^ | .899^**^ | .906^**^ | .939^**^ | .889^**^ | .889^**^ | .904^**^ | 1 |

Notes: ^a^ Mean of the total measures as well as each dimension on the measures was applied in the Pearson correlation tests.

^b^ Codes: I1 for Unreadiness dimension on INDECISION Scale; I2 for Lack of self-knowledge dimension on INDECISION Scale; I3 for Lack of options knowledge dimension on INDECISION Scale; I4 for External complexity dimension on INDECISION Scale; I5 for Lack of decision-making competence dimension on INDECISION Scale; I6 for Negative mentality dimension on INDECISION Scale; IT for the total measure of INDECISION Scale. S1 for Readiness dimension on SIS; S2 for Information dimension on SIS; S3 for Identity dimension on SIS; S4 for Barriers dimension on SIS; S5 for Indecisiveness on SIS; S6 for Self-doubt dimension on SIS; ST for the total measure of SIS.

^c **^ means P <.001.

**Specialty Indecision Scale, 2nd Edition**

**This scale evaluates your concerns for indecision in choosing a specialty**

Click on the response that more closely represents how much you agree with the statement.

| Item | 1 (Does not describe me at all) | 2 (Does not describe me very well) | 3 (Describe me somewhat) | 4 (Describe me well) |
| --- | --- | --- | --- | --- |
| **Readiness** |  |  |  |  |
| I haven’t spent much time thinking about choosing a specialty. |  |  |  |  |
| My specialty will come to me in due time. |  |  |  |  |
| I don’t know much about the specialty I’m interested in. |  |  |  |  |
| I’m too busy with my studies to worry about choosing a specialty. |  |  |  |  |
| It’s too early for me to decide on a specialty. |  |  |  |  |
| **Information** |  |  |  |  |
| I can’t find adequate and reliable information about the specialties I’m interested in. |  |  |  |  |
| I don’t know who can help me make a decision. |  |  |  |  |
| I don’t know what kind of information I need to help me decide. |  |  |  |  |
| I don’t know where I can get guidance for choosing a specialty. |  |  |  |  |
| I don’t know how or where to find information about specialties. |  |  |  |  |
| **Identity** |  |  |  |  |
| I’m not sure what kind of lifestyle I want to live. |  |  |  |  |
| Before exploring specialties, I need to know more about my own interests and goals. |  |  |  |  |
| I don’t have reliable information about my interests, abilities, and goals. |  |  |  |  |
| I need a clearer sense of who I am. |  |  |  |  |
| Someone will tell me what to specialize in. |  |  |  |  |
| **Barriers** |  |  |  |  |
| I can’t afford to pursue my desired specialty. |  |  |  |  |
| The specialty I’m interested in doesn’t pay well enough. |  |  |  |  |
| Someone important to me doesn’t like the specialty I’ve chosen. |  |  |  |  |
| I see a lot of problems with the specialty I’m interested in. |  |  |  |  |
| I can’t find one specialty that pays well and lets me serve others. |  |  |  |  |
| **Indecisiveness** |  |  |  |  |
| I’m interested in several specialties, but haven’t found the perfect one. |  |  |  |  |
| I’ve identified some good specialty choices, but can’t decide among them. |  |  |  |  |
| I’m attracted to two quite different specialties. |  |  |  |  |
| I am of two minds about my specialty. |  |  |  |  |
| I like parts of many different specialties. |  |  |  |  |
| **Self-doubt** |  |  |  |  |
| There are so many choices I don’t know where to start. |  |  |  |  |
| I’m unsure about my ability to succeed in different specialties. |  |  |  |  |
| I don’t know what factors I need to consider in making this decision. |  |  |  |  |
| Thinking about choosing a specialty makes me anxious. |  |  |  |  |
| Making important decisions is always difficult for me. |  |  |  |  |
