# Supplementary material for: Development and Validation of an Instrument to Measure Career Decision-Making Challenges of International Medical Students in China
Source: Perspect Med Educ. 2024 Nov 22;13(1):572–84. doi: 10.5334/pme.1384 (PMC11583610; doi:10.5334/pme.1384)
Supplement: Supplementary Files. — Appendixes 1 to 9. [file pme-13-1-1384-s1.zip › pme-1384_li-s1/Appendix 7.pdf]

Appendix 7 Correlation coefficient matrix for INDECISION Scale and SIS (n=102)

	I1	I2	I3	I4	I5	I6	IT	S1	S2	S3	S4	S5	S6	ST
I1	1													
I2	.749**	1												
I3	.698**	.773**	1											
I4	.544**	.692**	.676**	1										
I5	.602**	.776**	.603**	.653**	1									
I6	.591**	.737**	.727**	.684**	.728**	1								
IT	.807**	.924**	.882**	.812**	.838**	.876**	1							
S1	.657**	.605**	.520**	.543**	.637**	.542**	.671**	1						
S2	.673**	.653**	.666**	.547**	.595**	.663**	.739**	.743**	1					
S3	.641**	.650**	.520**	.522**	.649**	.590**	.689**	.823**	.824**	1				
S4	.488**	.572**	.441**	.551**	.597**	.501**	.604**	.810**	.756**	.836**	1			
S5	.600**	.638**	.509**	.571**	.699**	.610**	.697**	.768**	.731**	.797**	.739**	1		
S6	.700**	.723**	.586**	.546**	.664**	.672**	.755**	.750**	.835**	.812**	.707**	.789**	1	
ST	.696**	.709**	.601**	.604**	.708**	.662**	.768**	.899**	.906**	.939**	.889**	.889**	.904**	1

Notes: [a] Mean of the total measures as well as each dimension on the measures was applied in the Pearson correlation tests.

[b] Codes: I1 for Unreadiness dimension on INDECISION Scale; I2 for Lack of self-knowledge dimension on INDECISION Scale; I3 for Lack of options knowledge dimension on INDECISION Scale; I4 for External complexity dimension on INDECISION Scale; I5 for Lack of decision-making competence dimension on INDECISION Scale; I6 for Negative mentality dimension on INDECISION Scale; IT for the total measure of INDECISION Scale. S1 for Readiness dimension on SIS; S2 for Information dimension on SIS; S3 for Identity dimension on SIS; S4 for Barriers dimension on SIS; S5 for Indecisiveness on SIS; S6 for Self-doubt dimension on SIS; ST for the total measure of SIS.

[c] ** means P <.001.

Specialty Indecision Scale, 2nd Edition

This scale evaluates your concerns for indecision in choosing a specialty
Click on the response that more closely represents how much you agree with the statement.

Item	1 (Does not describe me at all)	2 (Does not describe me very well)	3 (Describe me somewhat)	4 (Describe me well)
**Readiness**				
I haven't spent much time thinking about choosing a specialty.				
My specialty will come to me in due time.				
I don't know much about the specialty I'm interested in.				
I'm too busy with my studies to worry about choosing a specialty.				
It's too early for me to decide on a specialty.				
**Information**				
I can't find adequate and reliable information about the specialties I'm interested in.				
I don't know who can help me make a decision.				
I don't know what kind of information I need to help me decide.				

I don’t know where I can get guidance for choosing a specialty.				
I don’t know how or where to find information about specialties.				
**Identity**				
I’m not sure what kind of lifestyle I want to live.				
Before exploring specialties, I need to know more about my own interests and goals.				
I don’t have reliable information about my interests, abilities, and goals.				
I need a clearer sense of who I am.				
Someone will tell me what to specialize in.				
**Barriers**				
I can’t afford to pursue my desired specialty.				
The specialty I’m interested in doesn’t pay well enough.				
Someone important to me doesn’t like the specialty I’ve chosen.				
I see a lot of problems with the specialty I’m interested in.				
I can’t find one specialty that pays well and lets me serve others.				
**Indecisiveness**				

I'm interested in several specialties, but haven't found the perfect one.				
I've identified some good specialty choices, but can't decide among them.				
I'm attracted to two quite different specialties.				
I am of two minds about my specialty.				
I like parts of many different specialties.				
**Self-doubt**				
There are so many choices I don't know where to start.				
I'm unsure about my ability to succeed in different specialties.				
I don't know what factors I need to consider in making this decision.				
Thinking about choosing a specialty makes me anxious.				
Making important decisions is always difficult for me.				
