# Supplementary material for: Development and Validation of an Instrument to Measure Career Decision-Making Challenges of International Medical Students in China
Source: Perspect Med Educ. 2024 Nov 22;13(1):572–84. doi: 10.5334/pme.1384 (PMC11583610; doi:10.5334/pme.1384)
Supplement: Supplementary Files. — Appendixes 1 to 9. [file pme-13-1-1384-s1.zip › pme-1384_li-s1/Appendix 8.docx]

**Appendix 8** Test-retest correlation for all dimensions of INDECISION Scale (n=86)

| **INDECISION Scale dimension** | **Stage** | **Mean±Standard deviation** | **Pearson correlation coefficient** |
| --- | --- | --- | --- |
| Total measure | Test | 2.706±1.0616 | .831^**^ |
|  | Retest | 2.552±1.0620 |  |
| Unreadiness dimension | Test | 2.830±1.0575 | .706^**^ |
|  | Retest | 2.650±1.1587 |  |
| Lack of self-knowledge dimension | Test | 2.552±1.1796 | .759^**^ |
|  | Retest | 2.241±1.2292 |  |
| Lack of options knowledge dimension | Test | 2.794±1.1201 | .710^**^ |
|  | Retest | 2.727±1.1743 |  |
| External complexity dimension | Test | 2.787±1.0327 | .683^**^ |
|  | Retest | 2.601±1.0895 |  |
| Lack of decision-making competence dimension | Test | 2.581±1.1504 | .738^**^ |
|  | Retest | 2.454±1.1537 |  |
| Negative mentality dimension | Test | 2.712±1.1533 | .740^**^ |
|  | Retest | 2.654±1.2308 |  |

Notes: ^a^ Mean of the total measure as well as each dimension on the measure was applied in the Pearson correlation tests.

^b **^ means P <.001.
