# Supplementary material for: Development and Validation of an Instrument to Measure Career Decision-Making Challenges of International Medical Students in China
Source: Perspect Med Educ. 2024 Nov 22;13(1):572–84. doi: 10.5334/pme.1384 (PMC11583610; doi:10.5334/pme.1384)
Supplement: Supplementary Files. — Appendixes 1 to 9. [file pme-13-1-1384-s1.zip › pme-1384_li-s1/Appendix 8.pdf]

Appendix 8 Test-retest correlation for all dimensions of INDECISION Scale (n=86)

INDECISION Scale dimension	Stage	Mean±Standard deviation	Pearson correlation coefficient
Total measure	Test	2.706±1.0616	.831**
	Retest	2.552±1.0620	
Unreadiness dimension	Test	2.830±1.0575	.706**
	Retest	2.650±1.1587	
Lack of self-knowledge dimension	Test	2.552±1.1796	.759**
	Retest	2.241±1.2292	
Lack of options knowledge dimension	Test	2.794±1.1201	.710**
	Retest	2.727±1.1743	
External complexity dimension	Test	2.787±1.0327	.683**
	Retest	2.601±1.0895	
Lack of decision-making competence dimension	Test	2.581±1.1504	.738**

	Retest	2.454±1.1537	
Negative mentality dimension	Test	2.712±1.1533	.740**
	Retest	2.654±1.2308	

Notes: [a] Mean of the total measure as well as each dimension on the measure was applied in the Pearson correlation tests.

[b] ** means P <.001.
