# Supplementary material for: Development and Validation of an Instrument to Measure Career Decision-Making Challenges of International Medical Students in China
Source: Perspect Med Educ. 2024 Nov 22;13(1):572–84. doi: 10.5334/pme.1384 (PMC11583610; doi:10.5334/pme.1384)
Supplement: Supplementary Files. — Appendixes 1 to 9. [file pme-13-1-1384-s1.zip › pme-1384_li-s1/Appendix 9.docx]

**Appendix 9-1** Comparison of SIS dimension ratings between current and previous studies

| **SIS dimension** | **IMSs in China**  (Current study)  n=102 | **Medical students in the US**  (Richard et al., 2007)  n=1,207 | **Medical students in Korea**  (Lee S-y et al., 2022)  n=181 |
| --- | --- | --- | --- |
|  | 4-point Likert scale | 4-point Likert scale | 5-point Likert scale |
|  | Dimension subtotal Mean (Ranking) | Dimension subtotal Mean (Ranking) | Dimension item Mean^a^ (Ranking) |
| Indecisiveness | 12.59 (1st) | 12.82 (1st) | 3.16 (3rd) |
| Information | 12.07 **(2nd)** | 10.82 (3rd) | 3.13 (4th) |
| Readiness | 11.75 **(3rd)** | 10.11 (4th) | 3.01 (5th) |
| Identity | 11.67 (4th) | 8.34 (5th) | 4.12 (1st) |
| Self-Doubt | 11.61 (5th) | 11.51 (2nd) | 3.46 (2nd) |
| Barriers | 10.65 (6th) | 8.28 (6th) | 2.24 (6th) |

Note: ^a^ As this study reported the data as item means, we presented the data in its original format for consistency. Furthermore, as the mean scores and the number of students for each grade were reported, we used these values to estimate the mean scores across all grades for comparison purposes.

**Appendix 9-2** Comparison of IMSs’ ratings on six dimensions between the INDECISION Scale and the SIS (n=102)

| **SIS** | | **INDECISION Scale** | |
| --- | --- | --- | --- |
| **Dimension** | Dimension item Mean^b^  (Ranking) | **Dimension^c^** | Dimension item Mean^b^  (Ranking) |
| Indecisiveness | 2.52 (1st) | Lack of Decision-Making Competence (LDMC) | 2.41 (5th) |
| Information | 2.41 (2nd) | Lack of Option Knowledge (LOK) | 2.91 **(1st)** |
| Readiness | 2.35 (3rd) | Unreadiness (UR) | 2.75 **(2nd)** |
| Identity | 2.33 (4th) | Lack of Self-Knowledge (LSK) | 2.32 (6th) |
| Self-Doubt | 2.32 (5th) | Negative Mentality (NM) | 2.71 **(3rd)** |
| Barriers | 2.13 (6th) | External Complexity (EC) | 2.68 **(4th)** |

Note: ^b^ Item mean scores instead of dimension subtotal scores were used, due to the differing number of items in each dimension across the two scales.

^c^ The comparisons were organised based on the content comparability of each dimension across the two scales.
